# Cardiac sympathetic activity in chronic heart failure: cardiac ^123^I-*m*IBG scintigraphy to improve patient selection for ICD implantation

**DOI:** 10.1007/s12471-016-0902-y

**Published:** 2016-09-27

**Authors:** D. O. Verschure, B. L. F. van Eck-Smit, G. A. Somsen, R. J. J. Knol, H. J. Verberne

**Affiliations:** 1 Department of Nuclear Medicine, Academic Medical Center, University of Amsterdam, Amsterdam, The Netherlands; 2 Department of Cardiology, Zaans Medical Center, Zaandam, The Netherlands; 3 Cardiology Centres of the Netherlands, Amsterdam, The Netherlands; 4 Department of Nuclear Medicine, Noordwest Ziekenhuisgroep, Alkmaar, The Netherlands

**Keywords:** Cardiac sympathetic activity, ^123^I-*m*IBG scintigraphy, Heart failure, Prognosis, Implantable cardioverter defibrillator, Cardiac resynchronisation therapy

## Abstract

Heart failure is a life-threatening disease with a growing incidence in the Netherlands. This growing incidence is related to increased life expectancy, improvement of survival after myocardial infarction and better treatment options for heart failure. As a consequence, the costs related to heart failure care will increase. Despite huge improvements in treatment, the prognosis remains unfavourable with high one-year mortality rates. The introduction of implantable devices such as implantable cardioverter defibrillators (ICD) and cardiac resynchronisation therapy (CRT) has improved the overall survival of patients with chronic heart failure. However, after ICD implantation for primary prevention in heart failure a high percentage of patients never have appropriate ICD discharges. In addition 25–50 % of CRT patients have no therapeutic effect. Moreover, both ICDs and CRTs are associated with malfunction and complications (e. g. inappropriate shocks, infection). Last but not least is the relatively high cost of these devices. Therefore, it is essential, not only from a clinical but also from a socioeconomic point of view, to optimise the current selection criteria for ICD and CRT. This review focusses on the role of cardiac sympathetic hyperactivity in optimising ICD selection criteria. Cardiac sympathetic hyperactivity is related to fatal arrhythmias and can be non-invasively assessed with ^123^I-*meta*-iodobenzylguanide (^123^I-*m*IBG) scintigraphy. We conclude that cardiac sympathetic activity assessed with ^123^I-*m*IBG scintigraphy is a promising tool to better identify patients who will benefit from ICD implantation.

## Introduction

**Fig. 1:**
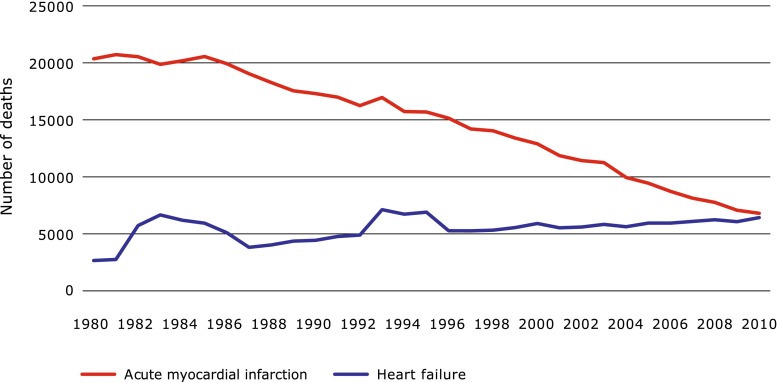
Number of deaths as a result of acute myocardial infarction and heart failure in the Netherlands from 1980 to 2010. The decrease in the number of deaths after myocardial infarction declines more rapidly than the increase in the number of deaths due to heart failure. Source: Centraal Bureau voor de Statistiek (CBS), the Netherlands

Heart failure (HF) is a life-threatening disease affecting approximately 26 million people worldwide [1]. The incidence of HF in the Netherlands ranges between 28,000 and 44,000 cases per year and increases with age; the majority of HF patients are older than 75 years [2]. Currently, there are between 100,000 and 150,000 patients with HF in the Netherlands. It is the only cardiovascular disease with both growing incidence and prevalence [3]. Reasons for this trend are related to increased life expectancy, improvement of survival after myocardial infarction and better treatment options for HF (Fig. 1). It is expected that the total number of HF patients in the Netherlands will increase to 275,000 in 2040 [4]. As a consequence, the costs related to HF care will increase: in 2007 these costs were 455 million euro which rose to 940 million in 2011 [2, 5]. For 2025, these costs are estimated at 10 billion euros [4].

Despite the successful introduction of treatment with a combination of beta-blockers and angiotensin-converting-enzyme inhibitors or angiotensin receptor blockers together with loop diuretics, the prognosis of chronic HF (CHF) remains unfavourable. The most recent European data (ESC-HF pilot study) demonstrate that 12-month all-cause mortality rates for hospitalised and stable/ambulatory HF patients were 17 and 7%, respectively [6]. The majority of these deaths are caused by progression of HF, lethal arrhythmia and sudden cardiac death. The use of implantable devices such as implantable cardioverter defibrillators (ICD) and cardiac resynchronisation therapy (CRT) has improved the overall survival of CHF patients [7–10]. Current European guidelines recommend ICD for primary prevention of fatal arrhythmias in CHF subjects with an ejection fraction <35% and symptomatic HF NYHA class ≥2 under optimal pharmacological therapy [11]. In addition, CRT is recommended in CHF patients who remain symptomatic in NYHA class ≥2 under optimal pharmacological therapy, with a left ventricular ejection fraction (LVEF) <35% and wide QRS complex (≥130 ms).

ICDs applied for primary or secondary (i.e. already proven ventricular arrhythmias) prevention reduce the relative risk of death by 20 %. However, analysis of the MADIT II (Second Multicenter Automated Defibrillator Implantation Trial) has shown that the absolute reduction of fatal events was only 5.6 % (19.8 to 14.2 %) [8]. In addition, the SCD-HeFT (Sudden Cardiac Death in Heart Failure Trial) study showed that the annual number of ICD shocks was 7.1 % of which 5.1 % were appropriate in the first year rising to 21 % in the 5th year post-implantation [12]. However, three years after ICD implantation for primary prevention, a remarkably high percentage of 65 % had never received appropriate ICD therapy. Moreover, there is also a risk of malfunction and operative complications, e.g. inappropriate shocks, infection.

Although the indications for CRT are well established, between one-quarter and one-half of patients who receive a CRT device are reported to show no response to the intervention. The MIRACLE (Multicenter InSync Randomized Clinical Evaluation) trial showed no therapeutic effect in 33 % of patients using a clinical composite score [13].

Last but not least is the relative high cost of these devices. Therefore, it is essential, not only from a clinical but also from a socioeconomic point of view, to optimise the current selection criteria for CRT and ICD for primary prevention aimed at better identification of patients who will benefit from implantation.

Currently one of the selection criteria for CRT and ICD implantation for primary prevention is an LVEF <35%. However LVEF assessed by cardiovascular magnetic resonance imaging (CMR) is significantly lower compared with echocardiography [14]. Therefore CMR would significantly increase the number of CHF patients eligible for CRT or ICD implantation. This illustrates that the method to assess LVEF has substantial impact on the selection of ‘appropriate’ patients for CRT and ICD implantation. The lack of uniformity among imaging modalities to assess LVEF raises the question if other parameters may be useful to better identify those patients who will benefit from CRT or ICD implantation. One of those alternative parameters might be cardiac sympathetic hyperactivity, which is related to poor prognosis and fatal arrhythmias in CHF. Cardiac sympathetic activity can be non-invasively assessed with cardiac ^123^I-*meta*-iodobenzylguanide (^123^I-*m*IBG) scintigraphy. Scholtens et al. described in a relatively recent review the possible role of ^123^I-*m*IBG to better select CRT candidates [15]. Therefore the role of ^123^I-*m*IBG to better select CRT candidates will only be briefly discussed in this review.

This review has three interrelated aims: First, to describe the pathophysiology of the cardiac sympathetic nervous system in HF. Secondly, how cardiac sympathetic activity can be non-invasively assessed with cardiac ^123^I-*m*IBG scintigraphy and thirdly to critically assess the available literature regarding cardiac ^123^I-*m*IBG scintigraphy in the specific context of ICD.

## Cardiac sympathetic activity

**Fig. 2:**
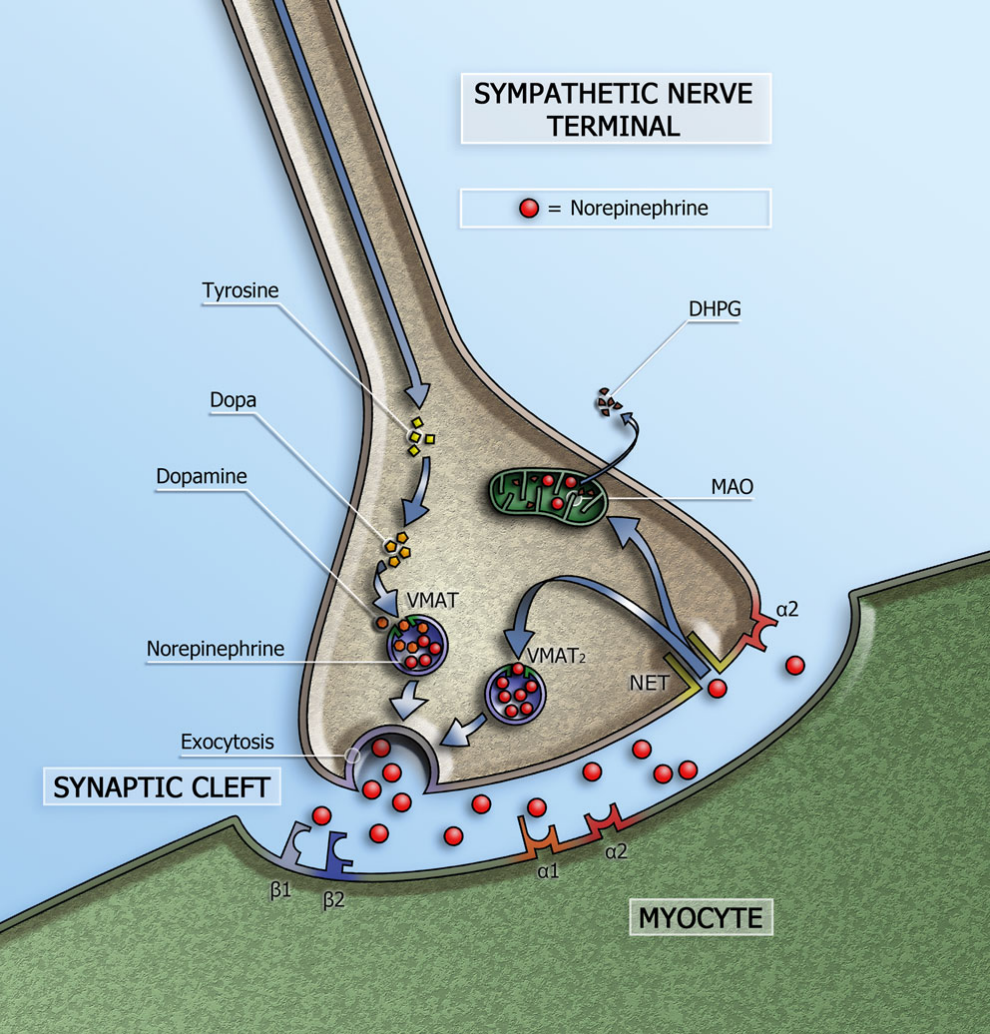
Schematic representation of the sympathetic synapse. Norepinephrine is synthesised within neurons by an enzymatic cascade. Dihydroxyphenylalanine (DOPA) is generated from tyrosine and subsequently converted to dopamine by DOPA decarboxylase. Dopamine is transported into storage vesicles by the energy-requiring vesicular monoamine transporter (VMAT). Norepinephrine is synthesised by dopamine β‑hydroxylase within these vesicles. Neuronal stimulation leads to norepinephrine release through fusion of vesicles with the neuronal membrane (exocytosis). Apart from neuronal stimulation, release is also regulated by a number of presynaptic receptor systems, including α2-adrenergic receptors, which provide negative feedback for exocytosis. Most norepinephrine undergoes reuptake into nerve terminals by the presynaptic norepinephrine transporter (NET) and is re-stored in vesicles (following uptake by vesicular amine transporter 2 (VMAT2)) or is metabolised in cytosol dihydroxyphenylglycol (DHPG) by monoamine oxidase (MAO)

Norepinephrine is the neurotransmitter of the cardiac sympathetic system and is stored in vesicles in the presynaptic nerve terminals (Fig. 2). On the basis of tissue norepinephrine content, the heart is characterised by dense sympathetic innervation with a gradient from atria to base of the heart and from base to apex of the ventricles [16]. Via exocytosis, norepinephrine is released into the synaptic cleft. Only a small amount of the released norepinephrine in the synaptic cleft is available to stimulate the post-synaptic β‑adrenergic receptors (β-AR) on the myocytes. Most of the norepinephrine undergoes reuptake into the nerve terminals via the uptake-1 mechanism. This transport system, i. e. norepinephrine transporter (NET), is sodium and chloride dependent and responsible for approximately 70–90 % of the norepinephrine re-uptake from the myocardial synaptic cleft.

The cardiac sympathetic system is one of the neurohormonal compensation mechanisms that plays an important role in the pathogenesis of CHF with impaired LVEF. Patients with CHF have increased cardiac sympathetic activity with increased exocytosis of norepinephrine from the presynaptic vesicles. In addition, the norepinephrine re-uptake via the NET in the sympathetic terminal nerve axons is decreased resulting in elevated synaptic levels of norepinephrine. Eventually this results in increased plasma and urinary levels of norepinephrine concomitant with the severity of left ventricular dysfunction [17–19]. Initially, β‑AR stimulation by increased norepinephrine levels helps to compensate for impaired myocardial function, but long-term norepinephrine excess has detrimental effects on myocardial structure and gives rise to a downregulation and decrease in the sensitivity of post-synaptic β‑AR [20, 21]. This downregulation leads to left ventricular remodelling and is associated with increased mortality and morbidity. Increased norepinephrine plasma levels are associated with poor prognosis in CHF [18]. However, these levels do not specifically reflect the sympathetic activity at a cardiac level. In addition, these measurements are time consuming and there is a high variability in measurements. However, cardiac sympathetic activity can be non-invasively visualised by nuclear techniques. To date, most commonly used tracers are norepinephrine analogues (^123^I-*m*IBG) for single photon emission tomography (SPECT) and ^11^C-hydroxyephedrine for positron emission tomography (PET). Both radiotracers are resistant to metabolic enzymes and show high affinity for presynaptic norepinephrine uptake-1 (NET) allowing the visualisation of presynaptic sympathetic nerve function. Other presynaptic PET tracers include ^11^C-epinephrine, ^11^C-phenylephrine, and ^18^F-LMI1195. ^11^C-CGP12177 is the most commonly used tracer for postsynaptic β‑ARs [22–24]. However, unlike ^123^I-*m*IBG, which can be centrally manufactured and then distributed, most PET agents are labelled with short half-life isotopes and are therefore only available in institutions with an on-site cyclotron. Although the early development of an ^18^F-labelled compound for sympathetic PET imaging is continuing [25], for the foreseeable future ^123^I-*m*IBG scintigraphy will remain the only widely available nuclear imaging method for assessing global and regional myocardial sympathetic innervation. In addition, cardiac ^123^I-*m*IBG scintigraphy is easily implemented in any department of nuclear medicine and thereby readily available for CHF patients.

## ^123^I-*m*IBG scintigraphy

^123^I-*m*IBG is a norepinephrine analogue that shares the same presynaptic uptake, storage and release mechanism as norepinephrine. Because ^123^I-*m*IBG is not metabolised, its accumulation over several hours is a measure of neuronal sympathetic integrity of the myocardium. Since the introduction of cardiac ^123^I-*m*IBG scintigraphy, parameters of ^123^I-*m*IBG myocardial uptake and washout have been shown to be of clinical value in many cardiac diseases, especially for the assessment of prognosis [26–29]. Cardiac ^123^I-*m*IBG scintigraphy has been established as a highly reproducible and feasible technique to evaluate the global and regional cardiac sympathetic function [30, 31].

### ^123^I-*m*IBG scintigraphy planar acquisition and analysis

**Fig. 3:**
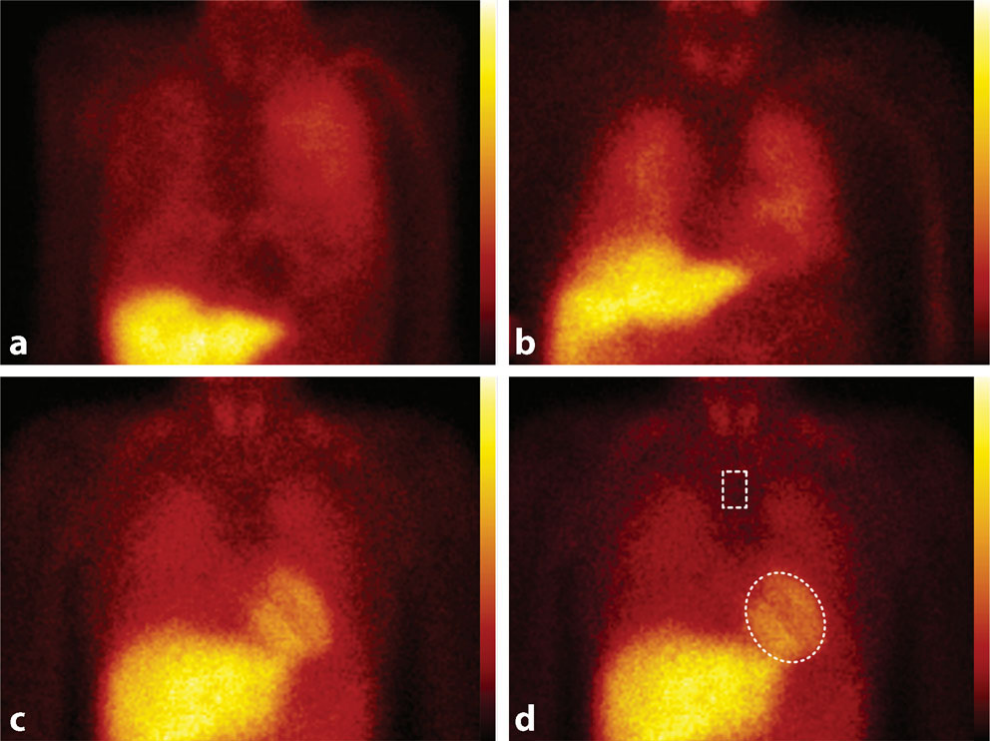
Two examples of planar cardiac ^123^I-*m*IBG scintigraphy using a medium collimator with different late (4h p.i.) myocardial ^123^I-*m*IBG uptake in subjects with the same LVEF compared with a healthy subject. **a** 63-year-old patient with ischaemic heart failure. **b** 68-year-old patient with ischaemic heart failure. **c** 32-year-old healthy person. **d** Example of placing a region-of-interest (ROI) over the heart (*H*) and fixed rectangular mediastinal ROI placed on the upper part of the mediastinum (*M*) for calculating H/M ratio

To block uptake of free ^123^I by the thyroid gland, subjects are pretreated with 250 mg of oral potassium iodide 30 min before intravenous injection of 185 MBq ^123^I-*m*IBG. Fifteen minutes (early acquisition) and 4 h (late acquisition) after administration of ^123^I-*m*IBG, 10-min planar images are acquired with the subjects in a supine position using a gamma camera equipped with a low energy high resolution or medium collimator. Based on the obtained planar (2D) images, three major outcomes of myocardial ^123^I-*m*IBG uptake can be determined: the early and late heart/mediastinal (H/M) ratio and cardiac washout rate (WO). The H/M ratio is calculated from planar ^123^I-*m*IBG images using a regions-of-interest (ROI) over the heart (Fig. 3). Standardised background correction is derived from a fixed rectangular mediastinal ROI placed on the upper part of the mediastinum [32]. The location of the mediastinal ROI is determined in relation to the lung apex, the lower boundary of the upper mediastinum, and the midline between the lungs. The H/M ratio is calculated by dividing the mean count density in the cardiac ROI by the mean count density in the mediastinal ROI [32]. The ^123^I-*m*IBG WO can be calculated using early and late H/M ratio (1). There are variations to the WO calculation using the myocardial count densities only, requiring a time-decay correction (factor of 1.21), without (2) or with background correction (3):

1 $$\text{WO}=\left \{\frac{\mathrm{(early H/M ratio)-}{(\mathrm{late H/M ratio)}}}{\mathrm{early H/M ratio}}\right \}\mathrm{*100}$$

2 $$\text{WO}=\left \{\frac{\mathrm{(early H)}-{(\mathrm{late H * 1.21)}}}{\text{early H}}\right \}\mathrm{*100}$$

3 $$\text{WO}=\left \{\frac{\mathrm{(early H - early M)}-{(\mathrm{late H - late M) * 1.21}}}{\mathrm{(early H - early M)}}\right \}\mathrm{*100}$$

The early H/M ratio predominantly reflects the integrity of sympathetic nerve terminals (i. e. number of functioning nerve terminals and intact uptake-1 mechanism). The late H/M ratio particularly offers information about neuronal function resulting from uptake, storage and release. The ^123^I-*m*IBG WO reflects predominantly neuronal integrity of sympathetic tone/adrenergic drive [33].

### ^123^I-*m*IBG scintigraphy SPECT acquisition and analysis

**Fig. 4:**
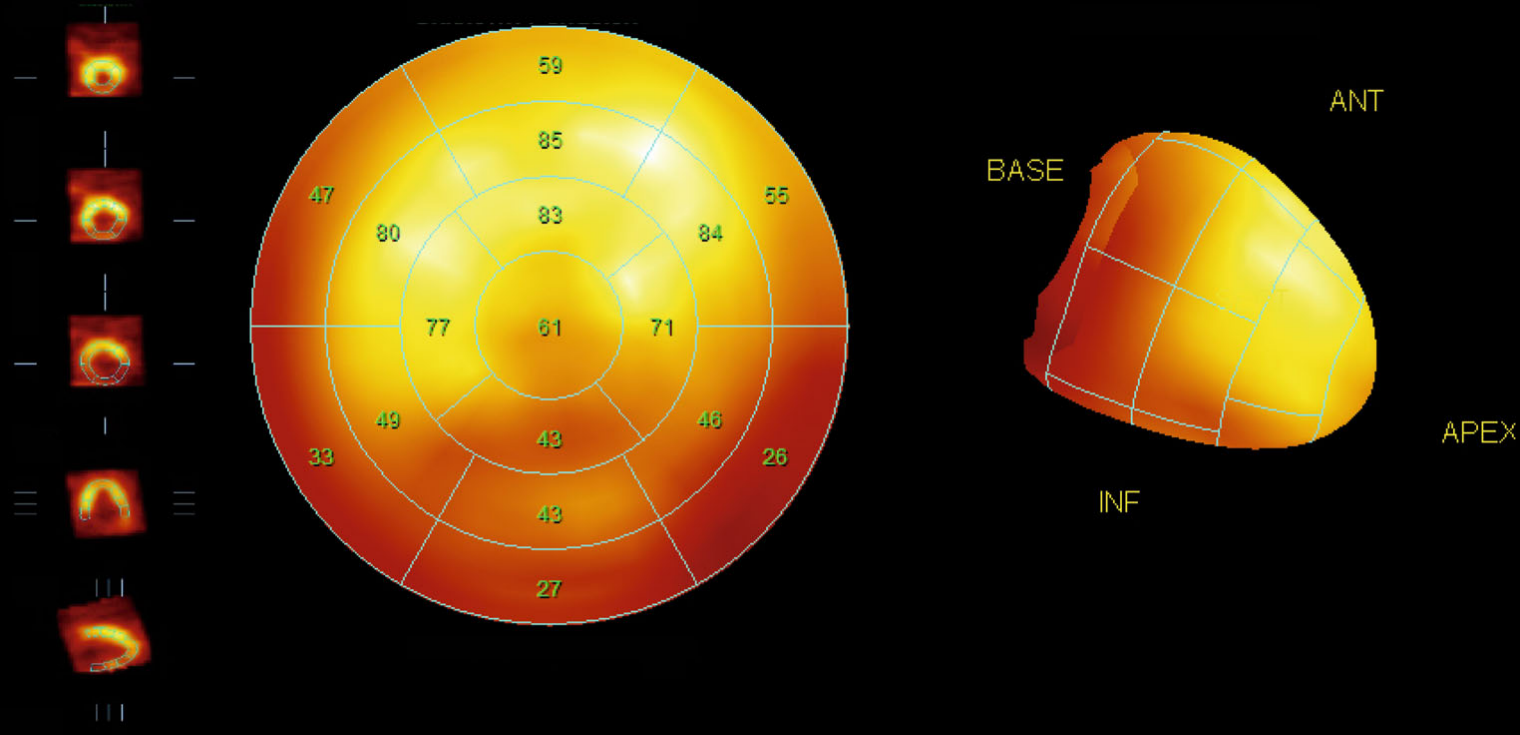
Example of late ^123^I-*m*IBG SPECT imaging. On the left the conventional short, vertical and horizontal axis, in the middle the corresponding 17-segment model polar map and on the right a 3D reconstruction. There is impaired regional ^123^I-*m*IBG uptake in the inferior wall from the myocardial base until the apex with extension to both inferoseptal and inferolateral regions

Further, compared with the H/M ratio derived from two-dimensional planar images, the results of three-dimensional imaging using SPECT provide a more complete understanding of global dysinnervation [34, 35]. Preclinical and animal studies suggested that myocardial regions with damaged or dysfunctional neurons but preserved perfusion can be a source of arrhythmias. Therefore, volumetric data such as SPECT may be of added value. The specific SPECT acquisition parameters have been described elsewhere but are largely comparable with those used for myocardial perfusion SPECT imaging [32]. Images can be processed and prepared for display and interpretation using the available commercial software packages (e. g. Emory Cardiac Toolbox and Cedar-Sinai Quantitative Perfusion SPECT). While there is no officially established method for scoring ^123^I-*m*IBG SPECT images, analysis can be performed similar to the conventional 17-segment/5-point model used for SPECT myocardial perfusion imaging (MPI) (Fig. 4; [36]). Therefore the ^123^I-*m*IBG SPECT images can easily be compared with MPI SPECT images in order to investigate the difference between regional innervation and possible myocardial perfusion abnormalities [37, 38].

### Planar ^123^I-*m*IBG as predictor of cardiac morbidity and mortality in CHF

Cardiac sympathetic hyperactivity is reflected by a decreased ^123^I-*m*IBG late H/M ratio and increased WO. Both are associated with increased fatal arrhythmia and cardiac mortality. It has been reported that late H/M ratio < 1.6 is associated with poor outcome [29]. However, meta-analyses showed that in patients with CHF, late H/M ratio is not only useful as a dichotomous predictor of events (high vs. low risk), but also has prognostic implication over the full range of the outcome value for all event categories except arrhythmias [39]. Although a WO cut-off value of 27 has been reported, it is important to realise that several other WO cut-off values have been reported [40]. Therefore there is no consensus on the exact WO cut-off value.

Initially ^123^I-*m*IBG scintigraphy assessed cardiac sympathetic activity in CHF has extensively been studied in small, single-centre studies. However the ADMIRE-HF study (ADreView Myocardial Imaging for Risk Evaluation in Heart Failure), a large multicentre, prospective study, reported that decreased late H/M ratio was associated with the composite endpoint of HF progression, ventricular tachyarrhythmia and death [29]. A total of 961 CHF patients with NYHA functional class II/III and a LVEF <35 % were enrolled. In this study, a predefined late H/M ratio cut-off value of 1.6 was used. Late H/M ratio was independent of brain natriuretic peptide and LVEF, as a predictor of the composite endpoint as well as of each individual component of the composite endpoint: progression of HF, lethal ventricular arrhythmia and sudden cardiac death. A late H/M ratio > 1.6 was associated with 1 % of cardiac deaths per year. In contrast, the annual cardiac mortality in a cohort with late H/M ratio < 1.2 was almost 10 times higher (9.6 %). In a re-analysis of the ADMIRE-HF data in 778 patients without an ICD at the start of the study, the predictive value of H/M ratio on ventricular tachyarrhythmia was analysed [41]. A late H/M ratio < 1.6 was associated with a 3.5-fold increased probability of an arrhythmia (hazard ratio [HR] 3.48, 95 % CI 1.52 to 8). This association was independent of other clinical predictors of ventricular tachyarrhythmia including LVEF. Therefore, it is tempting to speculate that ^123^I-*m*IBG scintigraphy may have a role in the management of HF patients. Indeed a prediction model for 5‑year cardiac mortality in patients with HF using ^123^I-*m*IBG has been developed [42]. The formula for predicting 5‑year mortality was created using a logistic regression model. By including the late H/M ratio in the model, the net reclassification improvement analysis for all subjects was 13.8 % (*p* < 0.0001). The inclusion of the late H/M ratio was most effective in the down reclassification of low-risk patients. To illustrate the independent prognostic role of myocardial ^123^I-*m*IBG scintigraphy some examples are given in Fig. 3.

### ^123^I-*m*IBG SPECT as predictor of cardiac morbidity and mortality in CHF

There is evidence that regional innervation/perfusion mismatch predispose to arrhythmias [43, 44]. Inhomogeneity in myocardial sympathetic innervation may create a myocardial substrate particularly vulnerable to arrhythmic death. This inhomogeneity can reflect sympathetic denervation from infarction, as well as reversible ischaemia. ^123^I-*m*IBG SPECT and ^123^I-*m*IBG/^99m^Tc-tetrofosmin SPECT mismatch was shown to be a predictor of appropriate ICD therapy [45, 46]. In 27 CHF patients with ICD for primary prevention ^123^I-*m*IBG SPECT and the ^123^I-*m*IBG/^99^mTc-tetrofosmin SPECT mismatch were independent predictors of arrhythmic events [45]. In a larger prospective study in 116 CHF patients, eligible for ICD implantation for both primary and secondary prevention of sudden cardiac death, SPECT was shown to be an independent predictor of appropriate ICD therapy and cardiac death [38]. The cumulative incidence of appropriate ICD therapy during 3 years of follow-up was significantly higher with a relatively large ^123^I-*m*IBG SPECT defect (median summed score ≥26). More recently, ^123^I-*m*IBG SPECT assessed cardiac sympathetic activity and ^99m^Tc-tetrofosmin assessed perfusion were studied as predictors of arrhythmic events in 471 patients with ischaemic CHF [47]. Multivariate proportional hazards analysis showed ^123^I-*m*IBG SPECT was an independent predictor of arrhythmic events (HR: 0.975, *p* = 0.042). In addition the authors concluded that the presumption of a monotonic increase in risk of an arrhythmic event with increasing ^123^I-*m*IBG SPECT defects may not be correct. This conclusion was based on the observation that in ischaemic CHF patients, those with intermediate defects appeared to be at the highest risk. A recent prospective PET study showed that ^11^C-hydroxyephedrine assessed sympathetic activity in ischaemic heart disease (*n* = 204), predicted cause-specific mortality from sudden cardiac arrest independently of LVEF and infarct volume [48]. These findings may be useful to better identify patients who, most likely, benefit from ICD implantation.

Recently the use of a computer quantitation method for myocardial ^123^I-*m*IBG SPECT studies was introduced [49]. In this study the incremental prognostic significance of ^123^I-*m*IBG SPECT imaging on all-cause and cardiac mortality for subjects (*n* = 938) in the ADMIRE-HFX study was assessed. The interactions between regional cardiac sympathetic activity (^123^I-*m*IBG SPECT) and abnormal perfusion (^99m^Tc-tetrofosmin SPECT) were explored. The highest cardiac mortality risk for ischaemic HF subjects (*n* = 619) was seen with perfusion defects involving 20–40 % of the myocardium. By comparison, non-ischaemic HF subjects (*n* = 319) with smaller perfusion abnormalities (<20 % of myocardium), but with a large discrepancy between ^123^I-*m*IBG and ^99m^Tc-tetrofosmin defect sizes, were at highest risk of cardiac death. The authors concluded that automated derived ^123^I-*m*IBG SPECT in combination with ^99m^Tc-tetrofosmin SPECT scores can effectively be used in the assessment of the prognosis of patients with HF.

### ^123^I-*m*IBG as predictor of CRT response

CRT has been shown to reduce mortality and morbidity due to reverse remodelling. The review by Scholtens et al., including nine studies with a total number of 225 patients, focused on CRT and cardiac innervation scintigraphy with ^123^I-*m*IBG. First and foremost there was a lack of uniform response criteria for CRT [15]. Nevertheless, in all available studies, ^123^I-*m*IBG showed positive changes in cardiac sympathetic nerve activity in responders to CRT. Additionally, ^123^I-*m*IBG imaging proved to be promising in identifying patients who did not benefit from CRT, alone or as part of an algorithm in combination with other parameters. However, studies are relatively small and extrapolation of these findings is hampered by the lack of uniform response criteria. Therefore, larger studies with uniform CRT response criteria are needed to confirm these provisional data.

### Need for standardisation

Although planar ^123^I-*m*IBG scintigraphy is a highly reproducible technique to assess cardiac sympathetic activity and has a small inter- and intra-observer variation [30], standardisation of acquisition and analysis is needed. The lack of standardisation between different institutions is one of the factors that have hampered the wide scale clinical implementation of cardiac ^123^I-*m*IBG scintigraphy. Some serious international efforts have been made to harmonise and standardise cardiac ^123^I-*m*IBG scintigraphy [32]. These recommendations include proposals for patient preparation, administered amounts of ^123^I-*m*IBG activity (MBq), scanning parameters (e. g. choice of collimators), and analysis of the acquired data to obtain the most used semi-quantitative parameters (i. e. early and late H/M ratio and ^123^I-*m*IBG WO). To further the role of ^123^I-*m*IBG scintigraphy in CHF, a strict use of these recommendations is essential to compare data from different institutions.

Incorporating ^123^I-*m*IBG scintigraphy into the assessment of CHF patients eligible for ICD implantation was associated with a reduction in ICD utilisation of 21 % [50]. Consequently, the number needed to screen to prevent one ICD implantation is 5. Screening with ^123^I-*m*IBG scintigraphy will reduce the costs per patient by 5500 and 13,431 US dollars over 2 and 10 years, respectively. In comparison, no screening with ^123^I-*m*IBG scintigraphy results in losses of 0.001 and 0.040 life-years over 2 and 10 years, respectively.

## Conclusion

In conclusion, HF is a widespread disease with rapidly increasing prevalence and is characterised by increased cardiac sympathetic activity. This hyperactivity is related to the progression of HF, fatal ventricular tachyarrhythmia and mortality. Non-invasive assessment of cardiac sympathetic activity with ^123^I-*m*IBG scintigraphy with standardised quantification techniques enables better identification of HF patients who will benefit from ICD implantation to improve their prognosis. Better selection of the CHF patient who will benefit from expensive HF therapy (i. e. CRT or ICD) will help to constrain the HF related costs.
